# Comparing Erythropoietin, Darbepoetin and No Erythropoiesis-Stimulating Agent Treatment in Preventing Anemia of Prematurity: A Multi-Center Retrospective Study

**DOI:** 10.3390/jcm15218251

**Published:** 2026-10-23

**Authors:** Noa Gadassi, Sivahn Goldstein, Michal Wulkan, Eynit Grinblatt, Dotan Shaniv, Shay Teitlboim, Calanit Hershkovich Shporen

**Affiliations:** 1 Neonatal Intensive Care Unit, Kaplan Medical Center ( Clalit Health Services), Rehovot 76100, Israeldotansh21@clalit.org.il (D.S.);; 2 Faculty of Medicine, Hebrew University of Jerusalem, Jerusalem 9112102, Israel; 3 Neonatal Intensive Care Unit, Meir Medical Center ( Clalit Health Services), Kfar Saba 4428163, Israel; 4 Pharmacy Services, Kaplan Medical Center ( Clalit Health Services), Rehovot 76100, Israel

**Keywords:** anemia, prematurity, erythropoiesis, erythropoietin, darbepoetin, hematocrit

## Abstract

**Objectives:** To compare the effects of two different erythropoiesis-stimulating agents [ESAs] and no treatment on the number of blood transfusions and hematocrit [Hct] level. **Methods:** A retrospective study. Kaplan Medical Center provided data on ESA-treated infants, comparing erythropoietin [EPO] given per protocol until 2018 and darbepoetin [DPO] given from 2018 onwards. Meir Medical Center’s data formed the control group. Data regarding ESA treatment were extracted from a computerized prospective database. A comparison of Hct levels at birth, 14 days of age, 1 month and before discharge and number of blood transfusions received during hospitalization was performed. Mean and standard deviation or median and interquartile range were used for continuous variables and frequency tables for categorical variables. **Results:** In total, 248 premature infants born between 2016 and 2021 under 1250 g, who survived beyond two weeks of age, were included. No significant differences were found between the EPO and DPO groups in terms of number of blood transfusions or Hct values. The control group received more blood transfusions after two weeks of age [0.8 ± 1.5 vs. 0.5 ± 1.2, *p*-value = 0.02]. In the treated group, higher Hct values were observed at one month of age [29.9 ± 4.2 vs. 37.9 ± 5.3, *p*-value < 0.01] and at discharge [29.7 ± 3.8 vs. 34.1 ± 4.6, *p*-value < 0.01] compared to the control group. **Conclusions:** ESA treatment was associated with higher hematocrit levels, and no significant differences in efficacy were found between EPO and DPO.

## 1. Introduction

Term infants are born with high hematocrit [Hct] levels and low erythropoietin [EPO] levels. After birth, Hct levels decline gradually due to the short life span of fetal hemoglobin reaching a nadir around two months of age. Hemoglobin levels at the nadir are usually no lower than 100 g/L and are not accompanied by clinical symptoms such as tachycardia, and are therefore termed physiological anemia. In response to the drop in Hct levels, a rise in erythropoietin levels is seen, causing Hct levels to stabilize.

In premature infants, the decline in Hct levels is faster and more pronounced, reaching a nadir of around 80 g/L in premature infants weighing 1000–1500 g and around 70 g/L in those weighing less than 1000 g. In contrast to term infants, the fast decline in Hct levels in premature infants, especially extremely low-birth-weight [ELBW] infants born under 1000 g, is often symptomatic, necessitating treatment with blood transfusions. Therefore, it is not considered physiological or benign [1]. Symptoms of anemia may include tachycardia, tachypnea, slow weight gain, desaturation episodes necessitating oxygen therapy, pallor and a rise in lactate levels. The above symptoms may prolong hospital stay and inadvertently cause complications due to infections and the need for frequent intravascular line changes [2].

A few of the known physiological reasons for anemia of prematurity [AOP], especially in the ELBW infant group, include a decrease in iron transfer from mother to fetus, which takes place mostly during the third trimester, and a decrease in the formation of red blood cells, which takes place mostly in vitro [1]. Added to that are the short life span of fetal red blood cells, a smaller increase in EPO levels, and bone marrow activation in response to anemia, compared to adults [3]. Known physiological factors in addition to AOP include the multiple blood draws necessary during the premature infant’s hospital stay [1].

Up to the year 1990, the only known affective treatment for severe AOP was blood transfusions. In the past, that required exposing the infant to many blood donors. With time, and the realization that small amounts were needed for each transfusion, the method of a “single donor” became popular. Blood was drawn from the same unit of blood until it was finished or expired. By doing so, the number of blood donors the infant is exposed to is reduced substantially. Despite the use of the “single donor” method and the rigorous screening of donated blood, blood transfusions still hold a risk for contracting blood-borne infections [2]. A connection between the development of necrotizing enterocolitis within 24–48 h of receiving a blood transfusion has also been found in research [4].

In 1978, for the first time, research was published showing that EPO administration to adult patients with kidney failure caused an increase in Hct levels and a decrease in the need for blood transfusions [2]. In premature infants, EPO levels are lower than those in term infants and adults, causing the low Hct levels during the nadir [5], therefore providing biological reasoning for the use of EPO for the treatment of AOP [6]. In 1990, EPO was first used for the treatment of AOP in a research setting [7]. Since then, two EPO treatment protocols have been accepted: early treatment [<7 days old] and late treatment [>7 days old]. Early treatment has been proven to decrease the need for blood transfusions, although most infants received blood transfusion before the administration of EPO [8].

In 2020, a meta-analysis showed an increase in grade 3 and retinopathy of prematurity in early EPO treatment versus late. Furthermore, early EPO treatment did not reduce blood donor exposure due to the fact that most infants received blood transfusions before the start of early treatment [6]. In contrast, in 2020, a post hoc analysis of the Preterm Erythropoietin Neuroprotection Trial [PENUT]—a randomized clinical trial designed to test the safety and efficacy of erythropoietin as a neuroprotective agent—was performed. This time, it sought to determine whether RBC transfusions and donor exposures would be decreased in the erythropoietin-treated infants compared with placebo-treated control participants. They found that high-dose erythropoietin given in the first 24 h, followed by maintenance dosing, can be a safe and successful treatment strategy that results in fewer infants needing transfusions and an overall decrease in the number and volume of RBC transfusions, as well as donor exposures, in treated infants [9].

Darbepoetin [DPO] as an erythropoiesis stimulating agent [ESA] with a longer half-life enables a once-a-week administration, compared to the thrice weekly administration of EPO for the treatment of AOP. When comparing between EPO and DPO for the treatment of AOP, a decrease in the need for blood transfusion was seen with DPO without an increase in retinopathy of prematurity [10]. Follow-up research revealed higher cognitive functions at 3.5–4 years of age in the DPO-treated infants, demonstrating a neuroprotective quality to the treatment with DPO [11].

At present, the use of ESAs for the treatment of AOP is controversial. Some Neonatal Intensive Care Units [NICUs] do not administer ESAs, while others administer it regularly. At the Kaplan Medical Center NICU, we have been administrating ESAs regularly to premature infants born at 1250 g or less, starting from 14 days of age and up to 34 + 0 weeks corrected age. Up until 2018 we used EPO. Since 2018, accompanied by a change in unit protocol, we have been using DPO.

Our aim in this study was to compare the effects of the two different ESAs used in our NICU over the years, EPO and DPO, on the number of blood transfusions and Hct level. By collaborating with the Meir Medical Center NICU, which does not administer ESAs, we were able to compare ESA treatment to no treatment as well.

## 2. Materials and Methods

This was a retrospective cohort study. Data were extracted from a computerized prospective database and included 248 premature infants born between 2016 and 2021 under 1250 g, who survived beyond two weeks of age. The Kaplan Medical Center provided data on ESA-treated infants, comparing EPO (Eprex Protecs 1000IU/0.5 mL, Janssen Inc., Toronto, ON, Canada) given per protocol until 2018 and DPO (Aranesp 40 mcg/0.4 mL, Amgen Europe B.V., The Netherlands) given from 2018 onwards. The Meir Medical Center NICU does not administer ESA and has similar blood transfusion thresholds as the Kaplan Medical Center NICU, as shown in Appendix D and Appendix E, and therefore their data formed the control group. All protocols can be found in the Appendix A, Appendix B and Appendix C. Sample size was calculated using the expected Hct level difference with and without treatment.

As stated previously, EPO was given per protocol until 2018 at a dosage of 250 units/kg three times a week, starting from 14 days of age. At the beginning of 2018, the unit protocol was changed due to the literature showing that DPO is non inferior to EPO, with a better dosage regime. Therefore, from 2018, DPO was given per protocol at a dosage of 10 mcg/kg once a week.

We compared the treatment groups to the nontreatment group based on the following variables: gender, birth weight, gestational age in weeks, the need for Invasive or Non-Invasive ventilation and the results of the SNAPPE II and CRIB II scores. Demographic and clinical variables were summarized using mean and standard deviation or median and interquartile range for continuous variables, and frequency tables for categorical variables.

Respiratory distress syndrome [RDS] was diagnosed by a chest radiography consistent with RDS together with supplementary oxygen or mechanical ventilation therapy. Bronchopulmonary dysplasia [BPD] was diagnosed according to the criteria of Bancalari et al. [12] including clinical and radiologic features, together with the requirement for oxygen supplementation at 36 weeks post-menstrual age. The diagnosis of necrotizing enterocolitis [NEC] was made in the presence of clinical and radiologic features according to the criteria of Bell et al. [13], and only definite NEC [Bell stages 2 and 3] was included.

Continuous variables were tested for normality using the Shapiro–Wilk test and were compared to the control group using the Mann–Whitney test. Categorical variables were compared using the Pearson Chi-square test. The significance level for the Chi-square test was set at *p* value < 0.05. Standardized residuals were added to determine which category created a significant difference.

We used the STROBE reporting guideline to draft this manuscript, and the STROBE reporting checklist when editing.

There were no patients or members of the public involved in the design, conduct, reporting, or dissemination of our research.

## 3. Results

The study cohort included 248 infants born at Kaplan Medical Center and Meir Medical Center between the years 2016–2021. Included in the study were infants with a birth weight under 1250 g that survived for at least two weeks. In total, 52 infants were treated with EPO, 42 were treated with DPO, and 154 received no treatment.

No difference was observed between the three study groups as regards gestational age in weeks, gender, or birth weight, including categorization into the groups appropriate for gestational age [AGA], small for gestational age [SGA], and large for gestational age [LGA]. When comparing treated infants [EPO or DPO] to those not treated, no difference was found as regards clinical risk index for babies [CRIB II] and the score for neonatal acute physiology with perinatal extension score [SNAPPE II]. A difference was found in the SNAPPE II score between the EPO-treated and DPO-treated groups, with the SNAPPE II score higher in the EPO-treated group [35.6 vs. 26.6, *p* value = 0.03], which might mean that the infants in the EPO-treated group were in worse clinical shape. In contrast, no difference was observed in the need for invasive or non-invasive ventilation between the groups, which does not support the previous assumption [Table 1].

When comparing the number of blood transfusions received during hospitalization, no significant difference was found between the EPO- and DPO-treated groups or between the treated [EPO or DPO] and not-treated groups. The same was observed when taking into account only blood transfusions received before 14 days of age, when ESA treatment was started. In contrast, when comparing the treated and not-treated groups from 14 days of age onwards, a statistically significant difference was observed. The treated group received 0.5 ± 1.2 blood transfusions, while the not-treated group received 0.8 ± 1.5 blood transfusions [*p* value = 0.02]. As stated previously, blood transfusion protocols for the treated and not-treated groups were very similar in terms of their Hct thresholds for blood transfusion, and were similar to the recent restrictive blood transfusion recommendations published by Deschmann et al. in 2024 [14]. Most probably, there is no clinical significance in this difference [Table 2].

**Figure 1:**
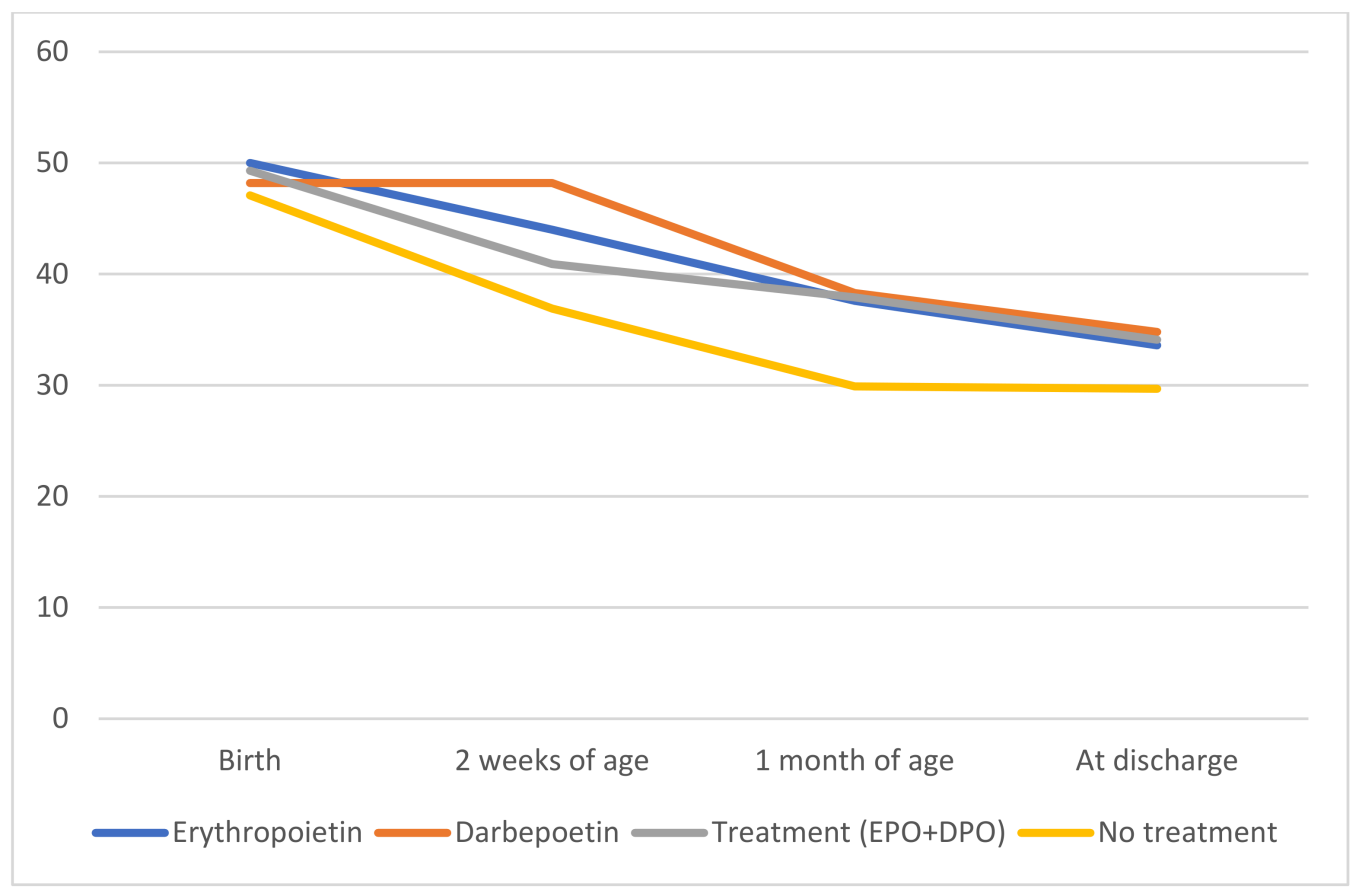
Hematocrit levels [%] over time.

We compared the effects of ESAs on Hct levels by looking at Hct levels at birth, at 14 days of age, at 1 month of age and before discharge. When comparing between EPO-treated and DPO-treated groups, no statistically significant difference was found, except at 14 days of age, before treatment with ESAs was begun. The DPO-treated group had lower Hct levels compared to the EPO-treated group [37 ± 6.6 vs. 44 ± 7.4, *p* value < 0.001]. In contrast, when comparing the treated and not-treated groups, a statistically significant difference was seen at 1 month of age [37.9 ± 5.3 vs. 29.9 ± 4.2, *p* value < 0.001] and at discharge [34.1 ± 4.6 vs. 29.7 ± 3.8, *p* value < 0.001]. A statistically significant difference was seen between these two groups at birth and at 14 days of age as well, but with time and treatment, the gap grew [Table 3, Figure 1].

When comparing possible known side effects of ESA treatment, such as retinopathy of prematurity and necrotizing enterocolitis, no statistically significant difference was found between the treated and not-treated groups. The same was seen when looking at common prematurity-associated comorbidities including intraventricular hemorrhage and respiratory distress syndrome. As for bronchopulmonary dysplasia [BPD], a statistically significant difference was found, showing a higher incidence of BPD in the treatment group compared to the no-treatment group [69.6% vs. 57%, *p* value = 0.05] [Table 4].

Our sample size was too small to reliably detect differences in these possible side effects, and therefore the greater rate of incidence of BPD should be viewed with caution, and warrants bigger studies to properly investigate this finding. What this comparison does show us is that no statistically significant differences were found between the EPO and DPO groups.

## 4. Discussion

This study was designed to compare between two ESA drugs administered to premature infants in order to prevent AOP, short-acting EPO and long-acting DPO. In addition, a comparison was made between ESA treatment [combining the EPO- and DPO-treated groups] and no treatment. The main variables compared were the need for blood transfusions during hospitalization and Hct levels.

In 2013, Ohls et al. showed that there were no significant differences in Hct levels, numbers of blood transfusions or comorbidities between treatments with EPO and with DPO [10]. Our study reinforces this conclusion and supports our department’s decision in 2018 to switch our ESA use from EPO to DPO. By doing so, we decreased the need for injections from 3 times a week to once a week, and therefore decreased the chance for injection site complications and pain exposure. Previous studies have shown that repeated pain exposure in premature infants negatively affects neurocognitive development [15] and chronic pain syndromes in adulthood [16].

Our main study limitation is the difference in Hct levels between the treated and not-treated groups, at birth and at 14 days of age. Despite the fact that CRIB II and SNAPP II scores, which reflect clinical severity during the first two days of life, were the same between the two groups, taking into account the differences in Hct levels at birth, a difference in clinical severity at birth between the two groups cannot be excluded. Added to this is the fact that iron supplementation in the not-treated group was started later in life due to differences in center protocols—a fact that might have had some impact on the difference in Hct levels between the treated and not-treated groups at 1 month of age.

Despite the difference in Hct levels from birth, when comparing Hct levels between the two groups at 1 month of age and discharge, an increase in the already existing difference can be seen. This shows that ESA treatment might benefit the infants not only by reducing blood transfusions but also by raising Hct levels.

Further limitations include the decline in admittances to the Kaplan Medical Center NICU over the years due to the opening of a new hospital nearby. The same decline was not seen at the Meir Medical Center, and may be the cause of the better clinical outcomes and higher Hct levels in the treatment group. Furthermore, comparisons between treated and untreated infants remain confounded by differences between the medical centers, calendar period, baseline hematocrit level, and iron supplementation, as stated previously. Our sample size was not calculated to evaluate the link between ESA treatment and the need for blood transfusions. A bigger sample size would most likely be required to do so properly. Originally, we tried to take into consideration whether prolonged cord clamping after birth was performed, and if so, for what duration. Information regarding cord clamping did not exist in the cohort of EPO-treated infants; therefore, we could not include it in our study. Lastly, we only included in the study infants that survived 14 days and beyond. This might have caused selection bias, but taking into consideration that ESAs were given only to infants from 14 days and up, this is the population that the treatment is relevant for.

## 5. Conclusions

We have shown that ESA treatment starting at 14 days of age is associated with higher Hct levels during hospitalization and at discharge, and might reduce the number of blood transfusions needed after the beginning of treatment. No significant differences in efficacy were found between EPO and DPO treatment.

In the future, our goal is to refine our patient selection for ESA treatment, treating only a select group with Hct levels below a preset value.

Follow-up research for our study cohort is needed to evaluate growth and developmental parameters. Doing so might help answer the question of whether higher Hct levels have an impact on growth and development, and reinforce the need for ESA treatment in the NICU.

## Figures and Tables

**Table 1:** Study group characteristics.

Parameter		EPO	DPO	*p* Value	No Treatment	Treatment EPO + DPO	*p* Value
No. patients		52	42		154	94	
Gender No. [Percentage]	Male	26 [50%]	17 [40.5%]	0.36	72 [46.8%]	43 [45.7%]	0.92
	Female	26 [50%]	25 [59.5%]		82 [53.2%]	51 [54.3%]	
Gestational age at birth [mean ± SD]		27.5 ± 1.8	27.6 ± 2.1	0.62	27.0 ± 2.5	27.6 ± 1.9	0.46
Weight at birth [grams] [mean ± SD]		973 ± 180.2	978.7 ± 220.4	0.68	943.2 ± 212.7	975.6 ± 198	0.5
AGA No. [Percentage]		47 [90.4%]	37 [88.1%]		119 [77.3%]	84 [89.4%]	
SGA No. [Percentage]		4 [7.7%]	3 [7.1%]		32 [20.8%]	7 [7.4%]	
LGA No. [Percentage]		1 [1.9%]	2 [4.8%]		3 [1.9%]	3 [3.2%]	
CRIB II score [mean ± SD]		8.9 ± 2.6	8.2 ± 3.1	0.14	8.7 ± 3.1	8.6 ± 2.8	0.78
SNAPPE II score [mean ± SD]		35.6 ± 22.4	26.6 ± 21.6	0.03	30.5 ± 21	31.6 ± 22.4	0.77
Invasive ventilation No. [Percentage]		31 [59.6%]	21 [50.0%]	0.35	80 [51.9%]	52 [55.3%]	0.61
Non-Invasive ventilation No. [Percentage]		46 [88.5%]	37 [88.1%]	0.956	135 [87.7%]	83 [88.3%]	0.88

EPO—Erythropoietin. DPO—Darbepoetin. AGA—Appropriate for gestational age. SGA—Small for gestational age. LGA—Large for gestational age. CRIB II—Clinical risk index for babies. SNAPPE II—Score for neonatal acute physiology and SNAP perinatal extension.

**Table 2:** Effects of erythropoiesis-stimulating agents on hematocrit levels and the need for blood transfusions.

		EPO [*n* = 52]	DPO [*n* = 42]	*p* Value	No Treatment [*n* = 154]	Treatment EPO + DPO [*n* = 94]	*p* Value
No. of blood transfusions	Total	1.9 ± 3	1.2 ± 2.1	0.32	1.7 ± 2.6	1.6 ± 2.6	0.83
	<2 weeks of age	1.4 ± 2	0.8 ± 1.3	0.14	0.9 ± 1.4	1.1 ± 1.7	0.28
	>2 weeks of age	0.5 ± 1.3	0.4 ± 0.9	0.43	0.8 ± 1.5	0.5 ± 1.2	0.02
Hct Levels [%]	1 month of age	37.6 ± 5.5	38.3 ± 5.1	0.32	29.9 ± 4.2	37.9 ± 5.3	<0.001
	At discharge	33.6 ± 4.5	34.8 ± 4.8	0.33	29.7 ± 3.8	34.1 ± 4.6	<0.001
Iron supplementation at discharge	3–4 mg/kg	44 [88%]	36 [90%]	0.39	67 [45.6%]	80 [88.9%]	<0.001
	5–6 mg/kg	6 [12%]	4 [10%]	0.39	80 [54.4%]	10 [11.1%]	<0.001

EPO—Erythropoietin. DPO—Darbepoetin. Hct—Hematocrit.

**Table 3:** Effects of erythropoiesis-stimulating agents on hematocrit levels.

		EPO [*n* = 52]	DPO [*n* = 42]	*p* Value	No Treatment [*n* = 154]	Treatment EPO + DPO [*n* = 94]	*p* Value
Hct Levels [%]	Birth	50.1 ± 7.7	48.2 ± 7.7	0.27	47.1 ± 6.2	49.3 ± 7.7	0.04
	2 weeks of age	44 ± 7.4	37 ± 6.6	<0.001	36.9 ± 6	40.9 ± 7.8	<0.001
	1 month of age	37.6 ± 5.5	38.3 ± 5.1	0.32	29.9 ± 4.2	37.9 ± 5.3	<0.001
	At discharge	33.6 ± 4.5	34.8 ± 4.8	0.33	29.7 ± 3.8	34.1 ± 4.6	<0.001
Age at start of iron supplementation [days]		16.3 ± 3.5	15.4 ± 1.4	0.1	33.8 ± 11.7	15.9 ± 2.8	<0.001

EPO—Erythropoietin. DPO—Darbepoetin. Hct—Hematocrit.

**Table 4:** Comorbidities and treatment complications.

		EPO [*n* = 52]	DPO [*n* = 42]	*p* Value	No Treatment [*n* = 154]	Treatment EPO + DPO [*n* = 94]	*p* Value
Total IVH		16 [30/8%]	6 [14.3%]	0.06	70 [45.5%]	22 [23.4%]	<0.001
IVH Grade 3–4		8 [15.4%]	3 [7.1%]	0.22	14 [9.1%]	11 [11.7%]	0.51
RDS		42 [80.8%]	30 [73.2%]	0.38	106 [68.8%]	72 [77.4%]	0.15
BPD		38 [73%]	26 [65%]	0.4	85 [57%]	64 [69.6%]	0.05
NEC		4 [7.7%]	2 [4.8%]	0.56	14 [9.1%]	6 [6.4%]	0.45
ROP		8 [15.7%]	10 [25%]	0.27	34 [22.8%]	18 [19.8%]	0.58
ROP Grade	1	1 [12.5%]	1 [10%]		20 [58.8%]	2 [11.1%]	
	2	5 [62.5%]	4 [40%]		12 [35.3%]	9 [50%]	
	3	2 [25%]	5 [50%]		2 [5.9%]	7 [38.9%]	

EPO—Erythropoietin. DPO—Darbepoetin. IVH—Intraventricular hemorrhage. RDS—Respiratory distress syndrome. BPD—Bronchopulmonary dysplasia. NEC—Necrotizing enterocolitis. ROP—retinopathy of prematurity.

## Data Availability

The dataset generated and analyzed during the current study is not publicly available due to patient confidentiality and data protection restrictions. However, de-identified data are available from the corresponding author, S.G., upon reasonable request and with appropriate institutional approval.

## Abbreviations

The following abbreviations are used in this manuscript:

AGA	Appropriate for gestational age
AOP	Anemia of prematurity
BPD	Bronchopulmonary dysplasia
CRIB II	Clinical risk index for babies
DPO	Darbepoetin
ELBW	Extremely low birth weight
EPO	Erythropoietin
ESAs	Erythropoiesis-stimulating agents
Hct	Hematocrit
IVH	Intraventricular hemorrhage
LGA	Large for gestational age
NEC	Necrotizing enterocolitis
NICU	Neonatal intensive care unit
RDS	Respiratory distress syndrome
ROP	Retinopathy of prematurity
SGA	Small for gestational age
SNAPPE II	Score for neonatal acute physiology and SNAP perinatal extension

## Appendix A. Erythropoetin [EPO] and Darbepoetin [DPO] Treatment Protocols—Kaplan Medical Center NICU

Who to treat:

- Infants with a birth weight of less than 1250 g who receive enteral feeding, starting from the age of 14 days until they reach 34–36 weeks postmenstrual age, and Hct necessitating;
- In special cases, it can be given to infants with a higher birth weight or an older postmenstrual age, in order to avoid a blood transfusion;
- In the case that an infant is still hospitalized beyond 36 weeks postmenstrual age, treatment may be prolonged. Treatment most be discontinued before hospital discharge.

Treatment monitoring:

- Complete blood count including reticulocytes should be performed every 2 weeks. Absolute Reticulocyte Count [ARC] should be calculated.
  - If ARC is smaller than 200,000 after 10+ days of treatment, EPO dosage should be doubled.
  - The dosage of DPO should be corrected to the current weight according to ARC count.
- Blood pressure should be measured once a day during treatment duration.
- ARC = [%Reticulocytes × Red Blood Cell mass]/100.

EPO dosage: 250 units/kg given subcutaneously 3 times a week.
DPO dosage: 10 mcg/kg given subcutaneously once a week.

## Appendix B. Iron Supplementation Protocol—Kaplan Medical Center NICU

Indications: Every infant starting at 2 weeks of age or at the start of erythropoiesis-stimulating agent treatment, the earlier of the two.

Dosage: initial dose—3 mg/kg/day, may increase up to 6 mg/kg/day according to clinical need and infant’s tolerance of the supplement.

## Appendix C. Iron Supplementation Protocol—Meir Medical Center NICU

**Table A1:** Iron Supplementation Protocol—Meir Medical Center NICU.

Type of Feed	Breast Milk or Progestamil or Neocate	Breast Milk + Human Milk Fortifier	Formula for Premature Infants
Iron [1 drop = 2.5 mg]	5 mg/kg/day = 2 drops/kg/day Starting at 1 month of age and until iron enriched feeds	2.5 mg/kg/day = 1 drop/kg/day Starting at 1 month of age and until iron enriched feeds	No iron supplementation
Discharge recommendations	Iron supplementation [Ferippel drops] 2 drops/kg/day, Max 6 drops/kg/day, until 18 months of age.		

## Appendix D. Indications for Blood Transfusion—Term and Preterm Infants 14 Days Old and Onwards—Kaplan Medical CENTER NICU

1. Hct < 40% + need for >40% oxygen OR inotropic blood pressure support;
2. Hct < 35% + need for nasal ventilation support + need for <35% oxygen;
3. Hct < 25% + one of the following symptoms:
  ○ Tachycardia > 180 beats/min OR Tachypnea > 80 breaths/min for longer than 24 h;
  ○ Increase in oxygen needs compared to previous 48 h;
  ○ Weight increase of less than 10 g/day for the last 4 days [while receiving at least 100 cal/kg/day];
  ○ Increase in number and severity of apneic episodes and /or bradycardic episodes [over 10 resolving spontaneously episodes or over 3 episodes necessitating ventilation in the past 24 h];
4. Hct < 20% + Asymptomatic infant + Absolute reticulocyte count [ARC] < 100,000 reticulofyte/µLt.

Deviation from the above indications is possible, based on the decision of a senior physician, accompanied by documentation of the reason in the patient’s medical record.

## Appendix E. Indications for Blood Transfusion—Term and Preterm Infants—Meir MEDICAL CENTER NICU

Dosage: 15–20 mL/kg.

Clinical indications:

**Table A2:** Indications for Blood Transfusion—Term and Preterm Infants—Meir Medical Center NICU.

Mechanical Ventilation	Mechanical Ventilation	Mechanical Ventilation	Nasal Ventilation	Nasal Ventilation	Spontaneously Breathing	Spontaneously Breathing
<28 days	<28 days	>28 days	<28 days	>28 days	FiO2 > 21%	Room air
FiO2 > 30%	FiO2 < 30%	FiO2 > 21%				
Hct < 40%	Hct < 35%	Hct < 30%	Hct < 30%	Hct < 25%	Hct < 25%	Hct < 22.5%

Blood transfusion in emergency setting: O- blood type should be given.
